# A Case Report of Werner’s Syndrome With a Novel Mutation From India

**DOI:** 10.7759/cureus.8025

**Published:** 2020-05-08

**Authors:** Ajeet Singh, Satyaki Ganguly, Namrata Chhabra, Hitesh Yadav, Junko Oshima

**Affiliations:** 1 Dermatology, All India Institute of Medical Sciences, Raipur, IND; 2 Pathology, University of Washington, Seattle, USA

**Keywords:** werner syndrome, wrn gene, aging, progeria, novel mutation

## Abstract

Werner's syndrome (WS) or progeria adultorum is a heritable autosomal recessive disease in which the aging process is accelerated, just after puberty. It is caused by mutations in the WRN gene, which encodes a member of the RECQ family of DNA helicases and has a role in DNA repair. WS is being more appropriately recognized as a condition in which the lack of WRN protein results in an overall decline in the normal physiological functions of various organs rather than premature aging. Here, we describe a rare case of WS with a novel mutation from India. Our patient was an adult male with a history of growth arrest since puberty and other clinical features such as sclerodermatous skin changes, premature graying and thinning of hair, bilateral cataract, a single non-healing ulcer, hypothyroidism, underdeveloped secondary sexual characters with hypogonadism, infertility, squeaky voice, and early signs of arteriosclerosis. On genetic analysis, he was found to have a homozygous pathogenic variant c.3190C>T in exon 26 of the WRN gene, which has never been reported in WS.

## Introduction

Werner's syndrome (WS) or progeria adultorum is a heritable autosomal recessive disease in which the aging process is accelerated, just after puberty [1]. It is caused by mutations in the WRN gene, which encodes a member of the RECQ family of DNA helicases and has a role in DNA repair [2]. It is characterized by genomic instability and the premature onset of a number of age-related diseases such as senile appearance, ocular cataracts, dyslipidemia, diabetes mellitus, osteoporosis, arteriosclerosis, and malignancies [3]. Recently, WS is being more appropriately recognized as a condition in which the lack of WRN protein results in an overall decline in the normal physiological functions of various organs rather than premature aging. Here, we describe a rare case of WS with a novel mutation from India.

## Case presentation

**Figure 1:**
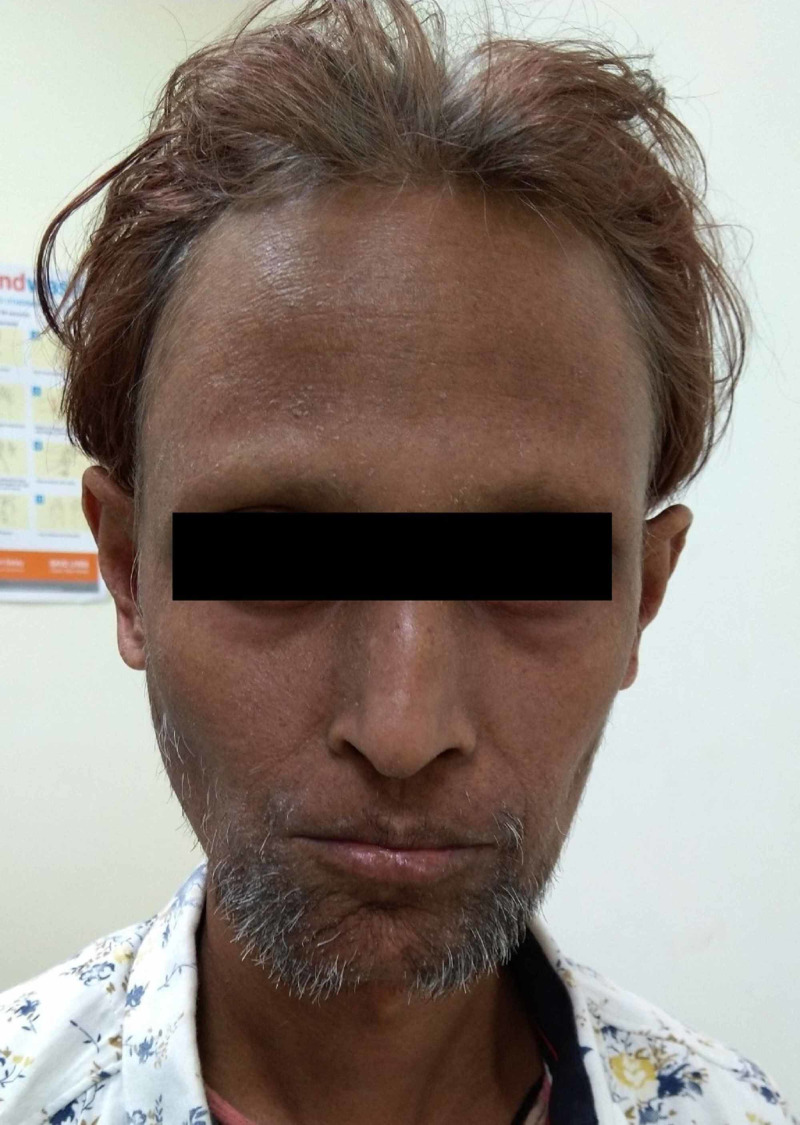
Androgenetic alopecia, thin and colored hair with graying evident near the scalp surface, aged appearance of the face, pursed lips, beaked nose, and sparse facial hair

A 38-year-old male presented with complaints of a non-healing ulcer over the left Achilles tendon region for four months. He also had associated complaints of dry skin and hoarseness of voice. The patient reported that he had normal body growth till the age of 12 years, following which there was no increase in weight and height and premature graying of hair started. His parents had a con-sanguineous marriage between first cousins. He was diagnosed with hypothyroidism at the age of 18 years, for which he has been under treatment since then. The patient also developed cataract in both eyes at the age of 25 years and underwent cataract surgery at that time. He also revealed a history of recurrent upper respiratory infections, and he had developed hoarseness of voice over 10 years since puberty. The patient was married for six years but did not have any offspring. Four months ago, he developed itchy papules over the left lower leg and left foot, and due to persistent itching, he developed an ulcer there. Clinical examination of the patient revealed an older appearance for his age, short stature, dry skin, thinning of hair, tight skin with subcutaneous atrophy, pursed lips with furrowing, high arched palate, squeaky and hoarse voice, beaked nose, palmoplantar hyperkeratosis, and flat feet (Figure 1).

**Figure 2:**
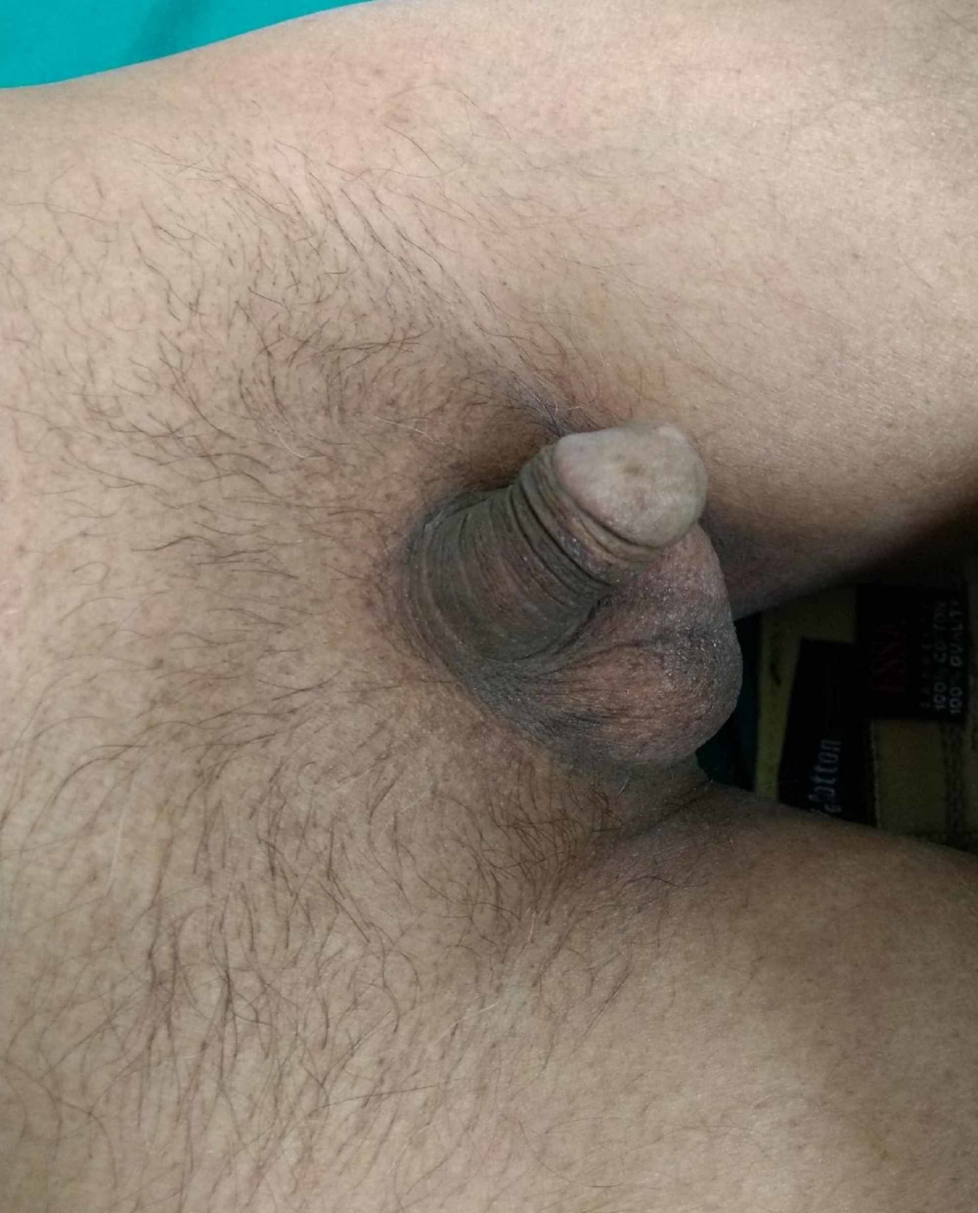
Sparse pubic hair with hypogonadism

Physical examination also revealed underdevelopment of secondary sexual characters with sparse facial, axillary, and pubic hair, and infantile genitalia with micropenis and decreased testicular volume (Figure 2).

**Figure 3:**
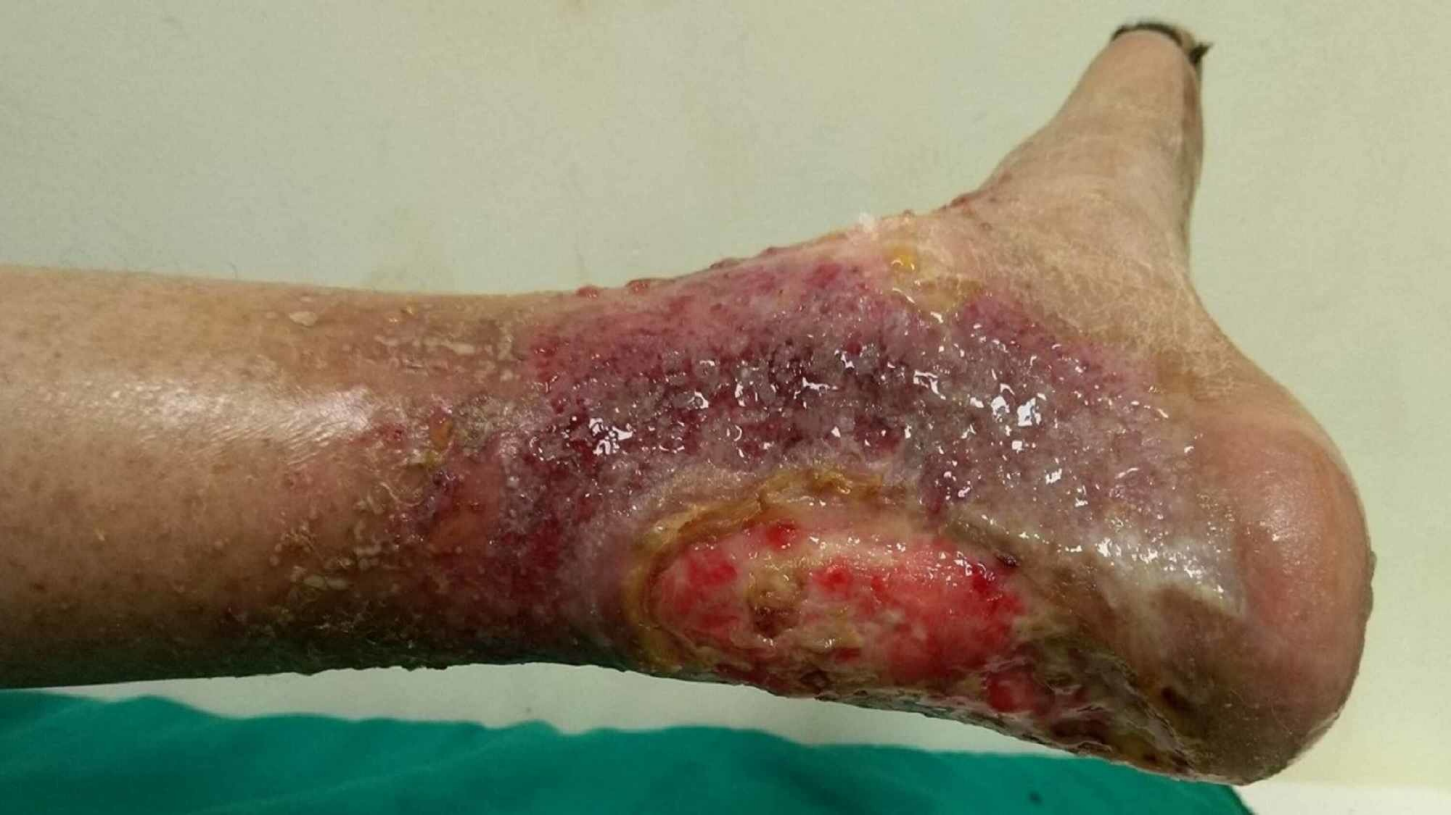
Well-defined ulcer with pale granulation tissue and hyperkeratotic margins, with the surrounding skin covered with serosanguinous discharge

There was a well-defined tender ulcer of size 6 x 8 cm, with hyperkeratotic margins present over the left Achilles tendon region. The floor of the ulcer had pale granulation tissue and slough with serosanguinous discharge. The surrounding skin was erythematous, suggestive of eczematous changes (Figure 3). 

**Figure 4:**
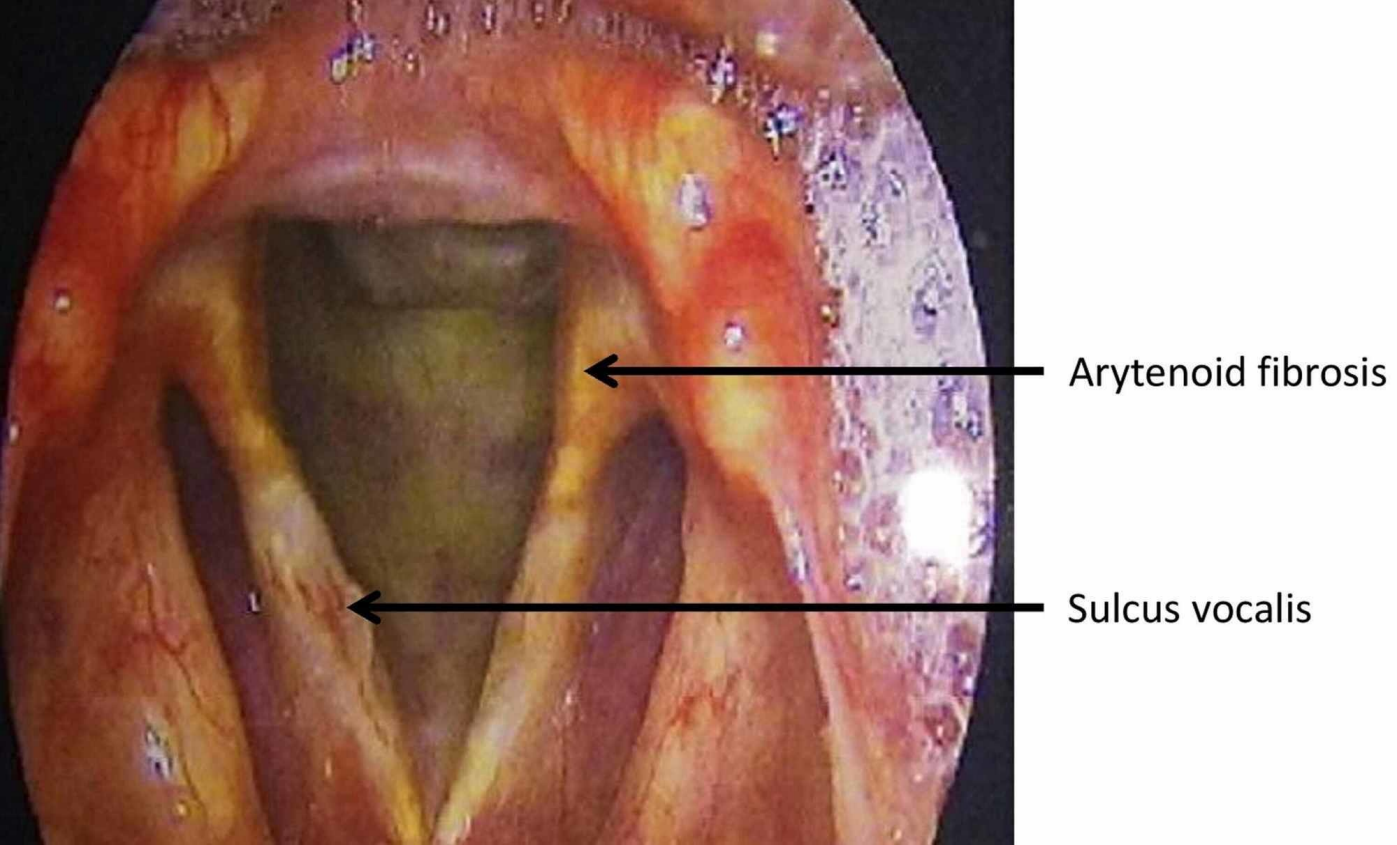
Laryngoscopy showing arytenoid fibrosis and sulcus vocalis

Ocular examination showed pseudophakia in both eyes, and slit-lamp examination showed multiple tessellations on the fundus. Routine investigations of the patient were within the normal limits. Hormone levels were within the normal limits except for elevated thyroid-stimulating hormone levels (26 mIU/L) and decreased total serum testosterone (135 ng/mL). Biopsy of the ulcer was performed, which showed no evidence of dysplasia/malignancy. Radiographic examination of hands and feet did not show any signs of osteosclerosis. Carotid Doppler showed early atheromatous changes in the right distal common carotid artery. Semen analysis performed in view of infertility showed decreased semen volume per ejaculate as well as decreased total sperm count. Laryngoscopy revealed fibrosis of the cricoarytenoid and sulcus vocalis (Figure 4).

Genetic analysis per the International Registry of Werner Syndrome revealed a substitution mutation with homozygous pathogenic variant c.3190 C>T in exon 26 of the WRN gene, which was confirmed by Sanger sequencing. The patient was counseled regarding his disease and possible long-term complications. He is under regular follow-up for an early detection of cutaneous malignancies and cardiovascular complications.

## Discussion

WS is an autosomal recessive disorder that was first described by a German physician, Otto Werner, in 1904, who reported four cases of siblings with symptoms and signs including juvenile cataract, scleroderma‑like changes in the extremities, short stature, premature aging of the face and gray hair, and hypogonadism [4]. WS has been reported in several populations, with a significantly high prevalence in Japan (estimated frequency of 1:100,000) and Sardinia province of Italy [5,6].

The sign first to be noticed is lack of growth spurt at puberty, which occurred in our case. Bilateral cataract together with a non-healing ulcer over the Achilles tendon region is a very frequent finding. Other clinical features of WS are skin atrophy, pinched face, gray and thin hair, hoarse voice, bilateral cataracts, diabetes, atherosclerosis, skin ulcers, hypogonadism, and osteoporosis. The most common cause of death is malignancy and myocardial infarction in the fifth decade of life [3]. The most typical neoplasms reported in WS patients are thyroid carcinomas (16%) followed by melanoma, soft tissue sarcomas, and leukemia [7]. Our patient was diagnosed with WS as per the diagnostic criteria proposed by the International Registry of Werner Syndrome (Table 1) [8].

**Table 1:** Application of the diagnostic criteria of the International Registry of Werner Syndrome in the present case

Cardinal Signs and Symptoms	Present Case
Cataract (bilateral)	Present
Short stature	Present
Characteristic dermatological pathology	Present
Parental consanguinity	Present
Premature graying and/or thinning of scalp hair	Present
Further signs and symptoms	
Diabetes mellitus	Absent
Hypogonadism	Present
Osteoporosis	Absent
Osteosclerosis of the distal phalanges of the digits	Absent
Soft tissue calcification	Absent
Voice changes	Present
Flat feet	Present
Evidence of premature atherosclerosis	Present
Any mesenchymal/rare/multiple neoplasms	Absent

Classical WS is caused by homozygous or compound heterozygous loss of function mutations within the WRN gene. The gene responsible for WS was discovered through the positional cloning method by Yu et al. in 1996 [9]. The WRN locus is found on human chromosome 8p12 and consists of 34 coding exons spanning 140 kb. It encodes for WRN protein, a 1,432-amino-acid-long, 163-kDa multifunctional nuclear protein with a 3'→5' exonuclease domain in its N-terminal region, an ATP-dependent 3'→5' helicase in its central region, and a nuclear localization signal in its C-terminal region [10,11]. The gene also contains consensus domains: the RecQ helicase conserved region (RQC) critical for initiating unwinding, and the "helicase, RNaseD, C-terminal conserved region" (HRDC) recruiting the WRN protein to double-stranded DNA breaks [12,13]. WRN is localized in the nucleolus and hence translocates at the positioning of DNA strand breaks. Available evidence indicates that WRN is involved in DNA replication, recombination and repair, telomere maintenance, transcription, mitochondrial function, and epigenetic modifications [14]. The WRN protein has an affinity for a few substrates (G4 quadruplexes, holiday junction, and bubble structures) in a proliferating cell, which helps in its role in DNA repair [1].

Up to the present, approximately 86 different mutations in WRN have been reported, which have been summarized in a review by Yokote et al. and other reports [15,16]. The majority of the mutations are stop codons that might result in loss of nuclear localization of WRN; as a result, mutated WRN protein is unable to enter the nucleus [17]. The WRN gene promotes genomic stability together with DNA repair; therefore, WS patients are predisposed to numerous cancers. Follicular carcinoma of the thyroid has particularly been related to C-terminal WRN mutations [18]. A recent study from Japan has reported three cases of WS with novel heterozygous variants together with Japanese founder mutation in the WRN gene [16]. Our patient has a substitution mutation with homozygous pathogenic variant c.3190C>T in exon 26 of the WRN gene, which was further confirmed by Sanger sequencing. This nucleotide substitution causes the premature termination of WRN protein translation at amino acid 1064 (p.Gln1064*). This variant is not one of the Indian founder mutations and has not been previously reported in WS patients [19].

At present, there are no specific treatments for WS. Many novel therapies are being explored, such as p38 mitogen-activated protein kinase inhibitors that have a role in aging and age-related diseases [20]. Symptomatic treatments for individual organ involvement are available. For non-healing cutaneous ulcers, aggressive management is required.

## Conclusions

Diagnosis of WS can be made clinically with the proposed International Registry of Werner Syndrome criteria, but genetic sequencing is recommended to find the association between any specific clinical feature or organ involvement and a genetic mutation, as has been reported previously for thyroid carcinomas in WS. Genetic sequencing can further enhance our knowledge regarding the role of various components of the WRN gene in the physiological process of aging.
